# Disorder-induced bulk photovoltaic effect in a centrosymmetric van der Waals material

**DOI:** 10.1038/s41699-023-00435-8

**Published:** 2023-11-21

**Authors:** Cheol-Yeon Cheon, Zhe Sun, Jiang Cao, Juan Francisco Gonzalez Marin, Mukesh Tripathi, Kenji Watanabe, Takashi Taniguchi, Mathieu Luisier, Andras Kis

**Affiliations:** 1 Electrical Engineering Institute, École Polytechnique Fédérale de Lausanne (EPFL), CH-1015 Lausanne, Switzerland; 2 Institute of Materials Science and Engineering, École Polytechnique Fédérale de Lausanne (EPFL), CH-1015 Lausanne, Switzerland; 3 Integrated Systems Laboratory, ETH Zürich, 8092 Zurich, Switzerland; 4 Research Center for Functional Materials, National Institute for Materials Science, 1-1 Namiki, Tsukuba, 305-0044 Japan; 5 International Center for Materials Nanoarchitectonics, National Institute for Materials Science, 1-1 Namiki, Tsukuba, 305-0044 Japan

**Keywords:** Nanophotonics and plasmonics, Two-dimensional materials

## Abstract

Sunlight is widely seen as one of the most abundant forms of renewable energy, with photovoltaic cells based on pn junctions being the most commonly used platform attempting to harness it. Unlike in conventional photovoltaic cells, the bulk photovoltaic effect (BPVE) allows for the generation of photocurrent and photovoltage in a single material without the need to engineer a pn junction and create a built-in electric field, thus offering a solution that can potentially exceed the Shockley–Queisser efficiency limit. However, it requires a material with no inversion symmetry and is therefore absent in centrosymmetric materials. Here, we demonstrate that breaking the inversion symmetry by structural disorder can induce BPVE in ultrathin PtSe_2_, a centrosymmetric semiconducting van der Waals material. Homogenous illumination of defective PtSe_2_ by linearly and circularly polarized light results in a photoresponse termed as linear photogalvanic effect (LPGE) and circular photogalvanic effect (CPGE), which is mostly absent in the pristine crystal. First-principles calculations reveal that LPGE originates from Se vacancies that act as asymmetric scattering centers for the photo-generated electron-hole pairs. Our work emphasizes the importance of defects to induce photovoltaic functionality in centrosymmetric materials and shows how the range of materials suitable for light sensing and energy-harvesting applications can be extended.

## Introduction

Exposing a crystal lacking inversion symmetry to light can result in a generation of photocurrent even at a zero-bias voltage due to the so-called bulk photovoltaic effect (BPVE)^1^, a second-order light-matter interaction. Compared to the conventional photovoltaic effect, which relies on the built-in electric field occurring at the interface between two different materials, BPVE occurs in a single material where photo-excited carriers are separated in real/momentum space due to the innate properties of the wavefunction geometry^2^. BPVE is an attractive mechanism for harvesting light energy because it is not restricted by the Shockley–Queisser efficiency limit^3^, and it has the potential to reach high conversion efficiencies in low-dimensional materials^4^. Nevertheless, these features of BPVE only apply to non-centrosymmetric materials, while centrosymmetric materials are devoid of BPVE due to the requirement of having a broken inversion symmetry^1^.

The modification of the crystal structure with external means can overcome this restriction and allow for the manifestation of BPVE in centrosymmetric materials by breaking the inversion symmetry^5^. This approach has so far been applied to van der Waals (vdW) layered semiconductors, externally activating BPVE via the use of a large strain gradient^6^, strain-induced polarization^7^, and reduced dimensionality^8^, enabled by their favorable mechanical properties. Despite the high BPVE coefficients of vdW materials achieved through this strain engineering^6,7^, device-related applications are limited by the need to form a hybrid structure with edges to exert local forces on the target material, which is prone to crack formation^6^. Alternatively, applying external electric fields is an effective way to break the inversion symmetry and realize BPVE^9,10^, but this requires large electric fields and ionic liquid gating, limiting the usefulness of this approach in practical applications.

One practical way of breaking the inversion symmetry is by introducing structural disorder. In two-dimensional (2D) materials, the most common form of structural disorder consists of point defects, giving rise to trap states for excited carriers^11^. Defects can also locally break the inversion symmetry, allowing for second-order harmonic generation (SHG) in centrosymmetric materials^12^. Yet, there is no clear experimental proof that BPVE, despite being the DC counterpart of SHG, can be similarly induced by structural disorder in centrosymmetric materials. Such a finding would be important since it would not only broaden the number of materials interesting for photovoltaic applications through defect engineering but also allow BPVE in a much simpler device scheme than the ones utilizing the mechanical deformation.

## Results

**Fig. 1:**
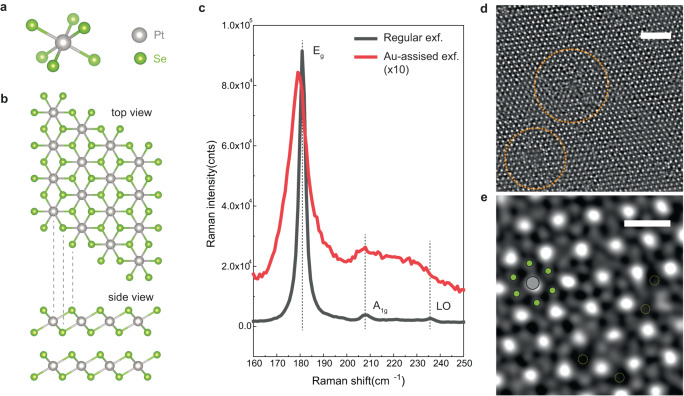
Defective PtSe_2_ crystal. **a**, **b** The unit cell (**a**) and top and side view of 1T-phase PtSe_2_ crystal structure (**b**). **c** Raman spectra of monolayer PtSe_2_ samples produced by regular exfoliation (black line) and Au-assisted exfoliation (red line). Dotted black lines indicate characteristic Raman peaks from the regular regular exfoliated sample that are related to E_g_, A_1g_, and LO phonon modes of 1T-phase PtSe_2_. Raman intensity from the Au-assisted exfoliation sample is multiplied by a factor of 10 for comparison. **d** STEM-HAADF image of a bilayer PtSe_2_ from Au-assisted exfoliation. The cluster-like defects are highlighted by orange dashed circles. The scale bar is 2 nm. **e** STEM-HAADF image with a small field of view. The green and gray dots represent Se and Pt atoms, respectively, and Se vacancies, displayed as dotted yellow circles, are visible. The scale bar is 5 Å.

PtSe_2_, a vdW layered material, is a suitable testbed for the proof-of-principle investigation of disorder-induced BPVE because of its inversion symmetry and its semiconducting nature in the few-layer form with a bandgap of 1.2 eV (0.4 eV) for the monolayer (bilayer) thickness^13^. The pristine PtSe_2_ crystal unit cell (Fig. 1a) is characterized by an octahedral coordination of Se atoms around Pt with a trigonal (T) unit cell. A PtSe_2_ monolayer (1 L) consists of one Pt layer sandwiched between two Se layers. The inversion symmetry of PtSe_2_ is preserved from monolayer to its bulk form due to AA vdW stacking (Fig. 1b). Its structure belongs to the centrosymmetric space group of ${\rm{P}}\bar{3}{\rm{m}}1$, with Se and Pt sites belonging to polar point groups C_3v_ and D_3d_, respectively. Recent studies suggest that point defects such as Se and Pt vacancies play an important role in PtSe_2_ to induce phenomena that are otherwise symmetry-forbidden in the pristine form, e.g., spin–orbit splitting^14,15^ and Rashba interaction^16^. Here, along the line of defect-induced properties found in this material, we provide evidence of the emergence of BPVE in semiconducting 2D PtSe_2_ due to the breaking of local inversion symmetry by structural disorder.

### Producing defective PtSe_2_ crystals

For this study, ultrathin 2D PtSe_2_ was produced using regular tape exfoliation (RE) and Au-assisted exfoliation (AE) technique, the latter of which is a method suitable for obtaining ultrathin large-area crystals^17^ (see Methods for more details). The structural quality of the produced crystals was first analyzed by Raman spectroscopy, as shown in Fig. 1c. Although both AE and RE samples show characteristic Raman peaks due to E_g_, A_1g_, and longitudinal optical (LO) phonon modes^18^, the intensity of the prominent E_g_ peak, due to the intra-layer in-plane vibration mode of top and bottom Se atoms, is significantly reduced in AE sample with respect to the RE sample. Also, compared to the RE sample, the E_g_ peak in the AE sample redshifts by ~1.5 cm^−1^ and shows a two-fold broadening. The origin of these Raman features can be attributed to the higher concentration of defects in the AE sample, possibly related to V_Se_, in analogy to the redshift and broadening observed for the E’ mode of monolayer MoS_2_ with sulfur vacancies^19^. In Supplementary Note 1, we further compare the redshift and broadening of the E_g_ peak between AE and RE samples and show that their difference gradually decreases with the number of layers with no noticeable difference for the bulk samples. This indicates that the structural defects are mostly likely occurring within the top few layers of AE PtSe_2_, which makes the Raman spectra of monolayer PtSe_2_ most sensitive to the presence of defects. Such defect formation could be due to sputtering by the incoming Au atoms during the deposition involved in the AE technique enabled by relatively low displacement threshold vacancy creation energy for chalcogen defects in PtSe_2_^20,21^. To support the hypothesis that the AE sample is physically damaged, we have reproduced the general features of the Raman spectrum associated with the AE sample by applying a mild plasma treatment on the RE sample (see Supplementary Note 2). Using aberration-corrected high-angle annular dark-field scanning transmission electron microscopy (HAADF-STEM) imaging, we directly visualize and confirm the presence of defects in AE PtSe_2_ (Fig. 1d, e). We can identify Se vacancies (V_Se_) as well as large cluster-like defects, both of which locally break the inversion symmetry of the pristine PtSe_2_ lattice. Thus, in the later text, we designate the AE (RE) sample as structurally defective (pristine).

### Polarization-dependent spontaneous photoresponse in defective PtSe_2_

**Fig. 2:**
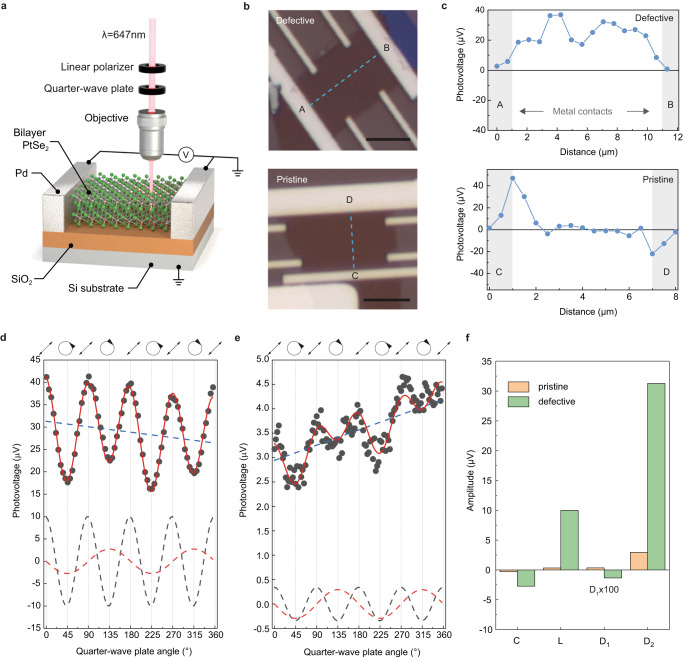
Spatial and polarization-dependent photovoltaic response in defective bilayer PtSe_2_. **a** Schematic of the setup for scanning photovoltage microscopy on a bilayer PtSe_2_ device. The optical excitation ($\lambda$ = 647 nm, P = 200 µW) is focused using a 50× objective, resulting in a laser spot area of ~0.7 µm^2^. The optical helicity is controlled by rotating the optical axis of a quarter-wave plate with an angle of $\theta$ with respect to the incident linear polarization direction (black line). **b** Optical micrographs of defective (top) and pristine (bottom) devices. The scale bars are 5 µm. **c** Photovoltage line scans from defective (top panel) and pristine (bottom panel) devices between the two measuring electrodes, along the dashed lines in **b**. **d**–**e** Photovoltage (black dots) as a function of $\theta$ for defective (**d**) and pristine (**e**) bilayer PtSe_2_. Data is obtained while laser illumination is centered between two measuring electrodes. Fitting from Eq. (1) is shown as a solid red curve. Dashed color lines represent three major components in the modulation indicated in Eq. 1; ${D}_{1}\theta +$ ${D}_{2}$ (blue, dashed line), ${\rm{C}}\sin (2\theta )$ (red, dashed curve), and ${\rm{L}}\sin (4\theta +\delta)$ (black, dashed curve). The state of the light polarization determined by the angle of the wave plate is labeled at the top of the graphs. **f** Amplitudes of fitting parameters ${\rm{C}}$, ${\rm{L}}$, ${D}_{1}$, and ${D}_{2}$ in Eq. 1, extracted from defective and pristine samples. ${D}_{1}$ values, which are accounted for experimental drift for both samples, are multiplied 100-fold.

To study the impact of defects on the photoresponse of PtSe_2_, we have performed polarization-controlled scanning photovoltage microscopy (Fig. 2a). Furthermore, we compare the photovoltaic response from multi-terminal devices made of defective and pristine bilayer PtSe_2_ on a SiO_2_/Si substrate (Fig. 2b). Firstly, the open-circuit voltage is simultaneously measured while scanning the laser along the central line connecting the two probing electrodes, with the profiles shown in Fig. 2c. The photovoltage from the pristine sample (Fig. 2c, bottom panel) is mainly generated at the electrode/PtSe_2_ interface with the opposite sign from the two interfaces, which can be attributed to the photovoltaic effect in the Schottky diode formed at the contact and the photothermoelectric effect at the semiconductor/metal junctions^22,23^. On the other hand, the interfacial photovoltaic response is relatively weak in the defective sample (Fig. 2c, top panel). Instead, the photovoltage increases when laser illumination occurs away from the electrodes and reaches its maximum when the spot is centered between the two electrodes, indicating that photovoltage is generated solely from defective PtSe_2_. Although both pristine and defective bilayer PtSe_2_ show gate-modulated source-drain current typical of semiconductors (see Supplementary Note 3), we observe a significantly lower on/off current ratio and linear *I–V* characteristics in the defective sample. These transport features indicate the metal-like character of ultrathin PtSe_2_ due to defect-induced mid-gap states^24^. This can also explain why photovoltage at the interface is not prominent in the defective sample: the photovoltaic effect is negligible due to a smaller Schottky barrier height as a result of Fermi-level pinning and defect states^25^.

To further investigate photovoltage generation under homogenous illumination on PtSe_2_, we control the light helicity using a quarter-wave plate (QWP) and compare the simultaneously measured photovoltage from the defective (Fig. 2d) and pristine (Fig. 2e) samples. We observe a much stronger photovoltage modulation and offset from the defective sample. A phenomenological expression for the photovoltage (${V}_{\rm{ph}}$) dependence on the polarization angle ${\rm{\theta }}$ can be written as:

1 $${V}_{\rm{ph}}={\rm{L}}\sin \left(4{\rm{\theta }}+{\rm{\delta }}\right)+{\rm{C}}\sin \left(2{\rm{\theta }}\right)+{D}_{1}{\rm{\theta }}+{D}_{2}$$

Here, ${\rm{L}}$ and ${\rm{C}}$ refer to the amplitude of the photovoltage modulated with $4{\rm{\theta }}$ and $2{\rm{\theta }}$-periodicity, which depends on linear and circular polarizations, respectively. The phase shift ${\rm{\delta }}$ is due to the initial linear polarization set by the linear polarizer. ${D}_{1}$ accounts for the sample drift during data acquisition and ${D}_{2}$ is a polarization-independent offset. Parameters ${\rm{L}}$, ${\rm{C}}$, ${D}_{1}$, and ${D}_{2}$ can be extracted from data by fitting to Eq. (1). We find larger amplitudes for all the parameters in the defective sample (Fig. 2f), with the exception of ${D}_{1}$ which accounts for sample drift. We observe that zero-bias photocurrent has an identical spatial distribution and polarization dependence as the photovoltage (see Supplementary Note 4). This supports the interpretation that both current and voltage responses originate from the homogenous illumination of PtSe_2_. The amplitudes of the linear and circular photocurrents increase linearly with the optical power, confirming the origin of the second-order response to the light electric field (see Supplementary Note 5).

The photon-drag effect (PDE) is an alternative nonlinear effect that can generate a similar polarization response as BPVE in 2D materials^26,27^. Since PDE requires a net in-plane photon momentum, it should be negligible in our measurement condition of normal incident light. Also, PDE alone cannot sufficiently describe the difference in photoresponse between defective and pristine PtSe_2_, as it can appear both in centrosymmetric and non-centrosymmetric materials. Yet, the requirement of broken inversion symmetry for BPVE is consistent with the structural disorder found in previous TEM and Raman analyses, making BPVE the most favorable explanation for our experimental data.

One possible explanation for BPVE induced by circularly polarized excitation, termed circular photogalvanic effect (CPGE), is Rashba-type splitting in the band structure caused by structural defects. As for pristine 1T-phase PtSe_2_, the bands are spin-degenerate because of the structural inversion symmetry. In the case of broken inversion symmetry, spin–orbit coupling can lead to spin-split bands in k-space with helical spin texture, the effect of which is called Rashba-type splitting. It is worth noting that the defect-induced Rashba effect was recently observed in both metallic and semiconducting PtSe_2_ from non-reciprocal charge transport^16^. Another phenomenon that is directly related to such splitting is the CPGE^9,28^. It can be attributed to the fact that the spin-split bands have different optical selection rules for left- and right-handed excitation, and for the opposite light helicity, photo-excited charge carriers flow reversely in these split bands. As a result, photocurrent changes its sign with light helicity.

For materials with high symmetry, including 1T-PtSe_2_ (D_3d_) and 3R-stacked transition metal dichalcogenides (C_3v_), CPGE should vanish at normal incidence due to symmetry-related arguments. Yet, it can occur if the symmetry of the material is reduced to a single mirror symmetry or even further^26^, which is expected in the presence of disorder. For defective PtSe_2_, the Se vacancy has C_3v_ symmetry, whereas the symmetry of cluster-like defects shown in Fig. 1d can be the lowest C_1_ symmetry without a single mirror symmetry. Therefore, Rashba-type splitting induced by such low-symmetry defects is expected to be responsible for CPGE in PtSe_2_. In this study, however, we focus on elucidating the origin of BPVE induced by linear polarization, which is the LPGE.

### First-principles calculations

**Fig. 3:**
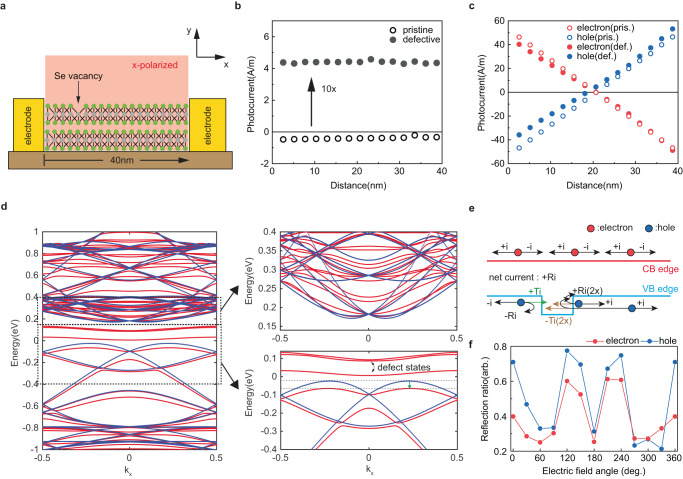
Ab initio simulation of photocurrent in defective PtSe_2_ bilayers from linear polarized light. **a** Simulated PtSe_2_ bilayer structure with a single Se vacancy introduced in the upper most Se layer. Linearly polarized light is shined over the entire structure. Here, the *x*-axis, which connects both device’s electrodes, is parallel to the armchair direction of PtSe_2_. **b**, **c** Spatial distribution of the photocurrent generated along the x-direction (**b**) and its electron and hole components (**c**) in pristine (empty circle) and defective (filled circle) PtSe_2_ with zero built-in potential. **d** Band structure of pristine (red) and defective (blue) bilayer PtSe_2_. The magnified insets show the energy levels near the conduction (top inset) and valence (bottom inset) band edges. The 40 meV bandshift of the top valence band edge is depicted as a green arrow. **e** Proposed photocurrent generation model for defective PtSe_2_. Se vacancies act as scattering centers for the photo-generated holes, but not for electrons. It is assumed that each photo-generated carrier has the same current probability to flow toward +*x* and −*x*, i.e., $\pm i$, since there is no applied electric field. For convenience, only three photo-generated electron–hole pairs are shown. Because of the induced potential barriers, holes have a probability *R* to be reflected against Se vacancies and *T* = 1 − *R* to be transmitted. Hence, the electron and hole currents become different (e.g., 3$i$ for electrons and −(3 − R)$i$ for holes in the left electrode), giving rise to a net current flow of R$i$ in defective PtSe_2_. **f** Electron and hole reflection probability as a function of their incident angle against the Se vacancies. 0 degree corresponds to carriers propagating along the armchair direction of PtSe_2_.

For that purpose, we apply an ab initio simulation approach combining density functional theory (DFT), maximally localized Wannier functions (MLWF), and the non-equilibrium Green’s function (NEGF) formalism (see Methods for more details) to explore light-matter interactions in bilayer PtSe_2_ with Se vacancies (Fig. 3a). Under linearly polarized illumination at a photon wavelength ${\rm{\lambda }}$_ph_ = 647 nm and zero built-in potential, the photocurrent flowing through the defective structure is at least one order of magnitude larger than in the pristine case (Fig. 3b). The overall photo-excited current is then decomposed into its electron and hole components that are plotted along a line connecting the device electrodes (Fig. 3c). In the pristine case, the electron and hole currents exactly compensate each other at every location. This electron–hole symmetry is broken by the presence of V_Se_ where the behavior of the hole current is more affected by scattering at the defect site than the electron one. This imbalance leads to a net non-zero photocurrent similar to what is observed in experiments.

**Fig. 4:**
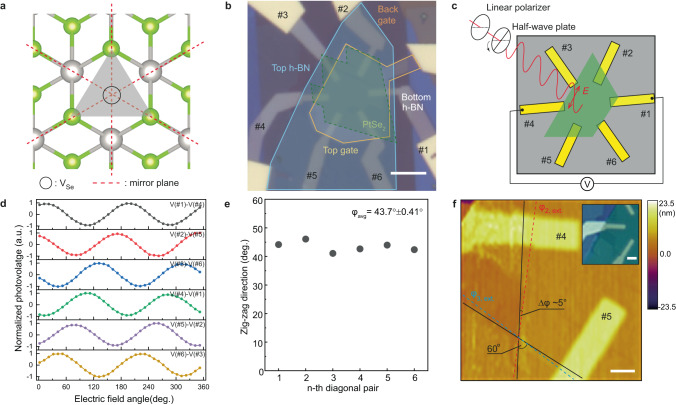
V_Se_-induced LPGE. **a** Top view of PtSe_2_ atomic structure with V_Se_ (black circle). V_Se_ has C_3v_ symmetry with broken inversion symmetry. A gray triangle represents a triangular wedge. **b** The optical micrograph of h-BN encapsulated defective bilayer PtSe_2_ with circularly oriented electrodes that are 60° apart. The optical micrograph of h-BN encapsulated defective bilayer PtSe_2_. The image is false-colored, highlighting different materials, and the electrodes are numbered for clarity. The scale bar is 5 µm. **c** Schematic of the photovoltage measurement scheme from diagonal electrode pairs. **d** Linear polarization-dependent photovoltage measured from six diagonal electrode pairs. LPGE responses are normalized to emphasize the constant phase shift. **e** Extracted angles (*φ*) of the zig–zag direction from six diagonal pairs. **f** 2D height profile by AFM around the electrodes #4 and #5. Black lines are the edge of PtSe_2_ identified from AFM, which is 60° apart. Blue and red dash lines are the extracted zig–zag directions. The scale bar is 1 µm. Inset is the optical micrograph of the same region of the device. The scale bar is 2 µm.

The electron–hole asymmetry can be explained by inspecting the band structures of pristine and defective PtSe_2_ bilayers, as calculated with DFT (Fig. 3d). It is found that the valence band (VB) is more altered by the presence of V_Se_ than the conduction band, with a downshift of the top VB by 40 meV, compared to the pristine case. Consequently, the V_Se_ creates local energy barriers that are important for holes and almost negligible for electrons. The reflection of holes against these potential barriers breaks the symmetry between the electron and hole currents, which induces a net current flow (Fig. 3e). Interestingly when incident electric field polarization is varied between 0° and 360° against V_Se_, the reflection coefficient of both holes and electrons exhibits a periodic behavior with a period of 120° (Fig. 3f). The reflection coefficient of holes is effectively higher than that of electrons, which in turn leads to the net flow of photocurrent being highly anisotropic (see Supplementary Note 6). This indicates that the V_Se_ behaves as a triangular scattering center whose properties can be described by a model based on a wedge-shaped potential lacking central symmetry^29^. Applied in various 2D systems for LPGE^30–32^, such a model commonly assumes that charged carriers, directed by alternating electric fields, are scattered by randomly located but identically orientated triangular wedges. If we picture these wedges to be at the position of each V_Se_ (Fig. 4a) that are randomly located, the scattering edges of each wedge are well aligned with one another since the PtSe_2_ crystal lattice maintains its 3-fold symmetry around V_Se_ as seen from the previously shown STEM image (Fig. 1e). Thus, V_Se_ meets the criteria to be the suitable atomic site for the wedges.

### Determining PtSe_2_ crystallographic direction from LPGE

Unlike for CPGE, there is no restriction for a trigonal symmetry prohibiting LPGE under normal incidence^32,33^. This motivates us to derive a phenomenological equation of LPGE with the second-order susceptibility based on the C_3v_ symmetry of V_Se_^34^, taking into account the experimental conditions, the position of probing electrodes, and the electric field of light, with respect to the crystallographic direction of PtSe_2_ with V_Se_ (see Supplementary Note 7 for the derivation). The normalized photovoltage from LPGE can be expressed as

2 $${V}_{\rm{LPGE}}=\sin 2\left({\rm{\alpha }}-\frac{3}{2}{\rm{\varphi }}+\frac{1}{2}{\rm{\kappa }}\right)$$

Here, ${\rm{\alpha }}$, *φ*, and ${\rm{\kappa }}$ are the electric field angle, the angle of the zig–zag direction of PtSe_2_, and the angle of the probing electrode, respectively, with reference to the linear polarization direction set by the initial alignment of the fast axes of the linear polarizer and the half-wave plate before its rotation. Since the LPGE phase in Eq. (2) is related to the zig–zag direction of PtSe_2_, we attempt to experimentally determine its crystallographic orientation to support our main argument that V_Se_ is the source of LPGE in PtSe_2_. We, therefore, realize a device with an array of electrodes placed along the edge of PtSe_2_ (Fig. 4b). We investigate the photovoltaic response measured from the diagonal pairs of electrodes while focusing the laser spot at the center of the PtSe_2_ flake and controlling its linear polarization (Fig. 4c). The photovoltage measured from each of the diagonal pairs is plotted as a function of the linear polarization angle (Fig. 4d). Data are normalized to visualize the continuous 30 degrees phase shifts introduced by sequentially measuring between diagonal pairs of electrodes in anti-clockwise order (in order of curves from top to bottom). Based on the $\frac{1}{2}{\rm{\kappa }}$ phase term in Eq. (2), the continuous 30° phase shift in Fig. 4d merely reflects the relative angle between neighboring electrodes, which is 60° and suggests ${\rm{\varphi }}$ to be a fixed value. Assuming an arbitrary ${\rm{\varphi }}$ when fitting each LPGE data with Eq. (2) produces a set of electrode angles showing the 6-fold symmetry of circularly oriented electrodes introduced by design (see Supplementary Note 8). By determining the electrode angles of the device from the reflection mapping (see Supplementary Note 9), we found from the fitting an average value $\bar{\varphi }$ = 43.7° (Fig. 4e).

The exfoliated layered materials commonly show sharp edges which belong to certain crystallographic orientations, the tendency of which is particularly strong for 1T-phase AA-stacked materials such as 1T-PtS_2_^35^, with their cleaved edges belonging to one of three zig–zag directions. We find that this is also the case for PtSe_2_, for which we find edges with a relative angle of 60° between electrodes #4 and #5, confirmed by AFM (Fig. 4f) and optical microscopy imaging (the inset of Fig. 4f). There is only around 5° difference between the angle of the sample edges and extracted zig-zag directions. Such a close match between optically identified and electrically extracted edge directions provides strong evidence that LPGE is induced by V_Se_ in PtSe_2_ and suggests the potential utility of LPGE for determining the crystal orientation of vdW crystals as an alternative to SHG.

## Discussion

In summary, our work has demonstrated BPVE induced by structural disorder in a centrosymmetric material, PtSe_2_. We have examined defective semiconducting bilayer PtSe_2_ with Raman spectroscopy and STEM, showing evidence of structural disorder introduced by Au-assisted exfoliation. While pristine PtSe_2_ exhibits a conventional photovoltaic response under zero-bias conditions, defective PtSe_2_ displays spontaneous photoresponse under homogenous illumination and generates LPGE and CPGE. We attribute the appearance of CPGE under normal incidence to the reduced crystal symmetry, possibly due to cluster-like defects in PtSe_2_. On the other hand, first-principle calculations capture our experimental findings on LPGE and suggest that Se vacancies act as asymmetric trigonal scattering sites to which photo-excited electrons and holes respond differently. Thus, disorder-induced BPVE in PtSe_2_ is related to two types of defects that have inherently different symmetries where the symmetry conditions for CPGE and LPGE are satisfied on a local scale. The wedge potential model applied to the atomic site of Se vacancies allowed us to determine the crystallographic orientation of PtSe_2_ experimentally. Our work extends the possible functionality of defects in centrosymmetric PtSe_2_ as a source for harvesting light energy and detecting light polarization and shows that defect engineering is a viable strategy for broadening the number of potentially interesting materials for BPVE-based photovoltaic applications.

## Methods

### Au-assisted exfoliation

The Au-assisted exfoliation was performed following the reported procedure^17^, which involves the evaporation of gold films on the bulk TMDC crystal. We first exfoliated bulk commercially available PtSe_2_ (HQ graphene) on a blue dicing tape (Nitto). Then, we evaporated 100 nm-thick gold film using an electron beam evaporator. Using thermal release tape (Nitto 3195MS), we peeled off the gold film, which would detach the topmost layers of bulk PtSe_2_, and transferred it to the SiO_2_/Si substrate at the releasing temperature of 120 °C. The substrate was treated with mild O_2_ plasma for 1 minute to remove the tape-related residue. The gold film was etched in an Au etchant, potassium iodide (KI), and iodine (I_2_) in DI water solution (1 g : 4 g : 40 g = I_2_: KI : H_2_O), for 5 minutes and then another 20 minutes in a new Au etchant solution. This is followed by a 5-min soaking in DI water to rinse away the Au etchant solution and a 30-min soaking in acetone and isopropanol (IPA) in order to remove any remaining polymer residues. As a result, ultrathin PtSe_2_ samples were obtained with a larger area than by the regular exfoliation method. The thickness of ultrathin PtSe_2_ was measured using atomic force microscopy (see Supplementary Fig. S1). We note that we do not observe residual Au layers/atoms on PtSe_2_ after removing Au by Au etchant based on EDX (see Supplementary Note 10).

### Raman spectroscopy

Raman spectroscopy measurement was performed at room temperature and atmospheric conditions using a confocal Renishaw inVia Confocal Raman microscope. A laser beam of 532 nm wavelength with a power of 1 mW was focused by an objective producing a Gaussian excitation area of 1 µm^2^ on target samples. To obtain high spectral resolution, 3000 lines/mm diffraction grating was employed for the Raman spectra of PtSe_2_.

### Scanning transmission electron microscopy

STEM imaging was performed using a Cs double aberration-corrected DCOR (CEOS) FEI Titan Themis. The Microscope is equipped with an X-FEG, Super-X EDX detector, and a Wein-type monochromator. STEM imaging experiments were done using the 80 kV primary acceleration voltage, probe convergence angle of 20 mrad, and the camera length was set to 185 mm, which corresponds to the HAADF detector 49–200 mrad collection angle. The estimated probe current was ~21 pA, and images were recorded using 1024 × 1024 pixels and 6 µs dwell times. The higher-order aberrations were minimized using the tableau. Velox software, ThermoFisher Scientific, and double Gaussian filtering in ImageJ were used to acquire and process the images. The position of individual Pt and Se atoms is clearly discernible by the image contrast where the intensity is directly *n*th proportional to the atomic number (*n* = 1.64–2), depending on the detector geometry.

### Device fabrication

#### Device #1 (bilayer PtSe_2_ on Si/SiO_2_ substrate)

The bilayer PtSe_2_ was produced by the Au-assisted exfoliation method explained above. The samples were spin-coated with PMMA polymer and put on a hot plate at 180 °C for 5 min. Electron-beam lithography was used to pattern the electrodes. Finally, an 80 nm Pd film was deposited by electron-beam evaporation for the electrodes, followed by a lift-off process using acetone to remove the PMMA layer.

#### Device #2 (bilayer PtSe_2_ encapsulated with h-BN)

The local metal gate (Cr 1 nm/Pt 5 nm) was fabricated using electron-beam lithography and electron-beam evaporation. Twenty-nanometre thick h-BN was used for the bottom gate dielectric. h-BN was produced by exfoliating it on a SiO_2_/Si substrate and then transferred on the prepared local metal gate by van-der Waals (vdW) pick-up transfer method using a polycarbonate (PC) film under atmospheric exposure. PC film is released at 170 °C, and the film is cleaned with Chloroform for 6 hours and then with isopropyl alcohol, followed by high vacuum (below 10^−5^ mbar) annealing at 340 °C for 6 hours. After the cleaning, the pre-contact electrodes (Pt 5 nm) were fabricated using electro-beam lithography and electron-beam evaporation. Bilayer PtSe_2_, produced by Au-assisted exfoliation on a SiO_2_/Si substrate, was picked up by 30 nm-thick top dielectric h-BN with PC film and released on the pre-contact electrodes with the same transferring and cleaning protocol as bottom dielectric h-BN. Finally, the top local gate (Pt 5 nm) and contact electrodes (Pd 80 nm) for the pre-contacts and local gates were fabricated using electro-beam lithography and electron-beam evaporation.

### Optoelectronic measurements

Scanning photocurrent and photovoltage microscopy was carried out with focused light excitation. We used 647 nm wavelength as an excitation source above the energy of the bandgap (0.4 eV) of bilayer PtSe_2_, which is focused into a Gaussian spot size (0.7 µm^2^) using a 50× objective while the excitation power was controlled by a neutral-density filter. As for the polarization control, the linear polarizer is used to set the initial polarization direction, and a quarter-wave plate was used to produce circular polarizations. It was replaced by a half-wave plate in the case of controlling the direction of linear polarization. Prior to rotating the wave plate, the fast axes of the linear polarizer and the wave plate are both aligned. The objective was mounted on an XY nano-positioner to control the excitation position on the sample, which was kept inside a high vacuum chamber (~10^−5^ mbar) during the measurement. The zero-biased photocurrent was measured by Keithley 2450 sourcemeter with no external voltage applied between two collecting electrodes. The photovoltage was measured in open-circuit conditions using an SR860 lock-in amplifier and a mechanical chopper (freq. 723 Hz), where the data acquisition was averaged for 1 s at the time constant of 300 ms. All the other electrodes in contact with PtSe_2_ not used for the electrical measurements were disconnected during the data acquisition.

### Ab initio simulations

The DFT calculations were performed with VASP^36^ within the generalized gradient approximation of Perdew, Burke, and Ernzerehof (PBE)^37^ using a $\Gamma$-centered Monkhorst–Pack *k*-point grid of dimension 21 × 21 × 1 and a plane-wave cutoff energy of 550 eV. The DFT-D3 method of Grimme^38^ was adopted to account for the vdW interactions. The PtSe_2_ bilayer structure was relaxed until the forces acting on each ion became smaller than 10^−4^ eV/Å. The DFT results were then transformed into a set of MLWF with the wannier90 code^39^. All the Wannier functions are well localized with a spread of less than 2.5 Å^2^. The MLWF Hamiltonian very accurately reproduces the DFT band structure. By applying an upscale technique on the hexagonal unit cell of the bilayer PtSe_2_, simulation domains of any size can be created, together with the corresponding Hamiltonian matrix and momentum operator. Here, we restricted ourselves to a 40 nm long slab between two semi-infinite leads that are treated as electrodes and collect the photo-generated electrons and holes. All photocurrents were simulated with a quantum transport solver relying on the NEGF formalism and dedicated electron–photon scattering self-energies (SSE) with ab initio inputs^40^. The electron-photon SSE can account for different polarization directions. Individual V_Se_’s were introduced by removing Se atoms from the top Se layer of the PtSe_2_ bilayers. To separate the electron and hole contributions to the photocurrent, we integrated the energy-resolved photo-excited current density within the energy range corresponding to the conduction and VBs, respectively. To map the directional dependence of the electron/hole reflection probability at V_Se_ scattering sites, we calculated the contribution to the electron and hole photocurrents for different carrier momentum directions. The reflection probability at V_Se_ scattering sites was deduced from the relative difference between the photocurrents of pristine and defective structures.

### Supplementary information

Supplementary information

## Data Availability

The data that support the findings of this study are available from the corresponding author upon reasonable request.
