# Supplementary material for: Disorder-induced bulk photovoltaic effect in a centrosymmetric van der Waals material
Source: NPJ 2D Mater Appl. 2023 Nov 21;7(1):74. doi: 10.1038/s41699-023-00435-8 (PMC11041738; doi:10.1038/s41699-023-00435-8)
Supplement: Supplementary file 1 — Supplementary information [file 41699_2023_435_MOESM1_ESM.pdf]

Table of Contents

Supplementary Note 1. AFM and Raman spectroscopy on RE and AE samples.......................2

Supplementary Note 2. Raman spectroscopy on the plasma-treated RE samples......................4

Supplementary Note 3. Charge transport in bilayer $PtSe_2$..4

Supplementary Note 4. Zero-biased photocurrent – polarization and spatial dependence.........6

Supplementary Note 5. Optical power *vs.* zero-biased photocurrent..8

Supplementary Note 6. Anisotropic photocurrent from *ab initio* calculation 8

Supplementary Note 7. Phenomenological LPGE equation..9

Supplementary Note 8. Extracting electrode angles from LPGE equation 13

Supplementary Note 9. Determining electrode angles and zig-zag directions of $PtSe_2$...........14

Supplementary Note 10 EDX analysis 15

References...15

Supplementary Note 1. AFM and Raman spectroscopy on RE and AE samples

The material source for $PtSe_2$ studied in this work is commercially available $PtSe_2$ from HQ graphene which is grown by charge vapor transport (CVT). We first used atomic force microscopy (AFM) to confirm the number of layers of exfoliated ultrathin $PtSe_2$ crystals by regular exfoliation (RE) and Au-assisted exfoliation (AE), based on the multiples of the unit monolayer thickness (Supplementary Figure 1). This was intended to compare the Raman modes of the samples at the same thickness. The thickness of monolayer $PtSe_2$ by RE (AE) is measured to be around 0.75nm (0.7nm), both of which are close to the reported values[1].

Raman spectroscopy was used to investigate the structural quality of $PtSe_2$. Supplementary Figure 2a shows the Raman spectra of monolayer (1L), bilayer (2L) and trilayer (3L) $PtSe_2$ obtained by RE where we observe three prominent Raman peaks showing 1T-phase $PtSe_2$: E_g, A_{1g} and longitudinal optical(LO) phonon modes[2]. E_g mode corresponds to intra-layer in-plane vibration of top and bottom Se atoms moving in the opposite directions, and A_{1g} mode is intra-layer out-of-plane vibration of top and bottom Se atoms moving in the opposite directions. LO mode consists of two vibrational modes, infrared active (A_{2u}) and Raman and infrared active (E_u).

In the case of 2L $PtSe_2$, we found the position of E_g, A_{1g} and LO modes to be around 179.7cm^{-1}, 207.4cm^{-1}, and 235.3cm^{-1}, respectively, consistent with the previous Raman analysis with the same excitation wavelength[1]. As thickness increases from 1L to 3L, the E_g peak redshifts from 180.9cm^{-1} to 179.05cm^{-1}, and the A_{1g} peak also redshifts from 208.43cm^{-1} to 207.61cm^{-1}. The red shift of the E_g peak was attributed to long-range Coulomb interaction[3], which has also been observed in other metal dichalcogenides (TMDCs) as well[4]. In bulk $PtSe_2$, E_g and A_{1g} peaks further redshift to 177.81cm^{-1} and 207.02cm^{-1}, respectively. Thus, the E_g peak shows a larger peak shift when the thickness is increased from monolayer to bulk. We note that the Raman peak of Si substrate was monitored as a reference. The Si substrate peaks from all the different samples were found to be within 520.26 ± 0.03cm^{-1}.

Interestingly, we found that the LO mode shows the largest intensity ratio with respect to the intensity of E_g mode at the 2L thickness and splits into two vibrational modes at the 3L thickness, merging into one broad peak above the 3L thickness. This anomalous behavior is not observed in ultrathin $PtSe_2$ grown by other methods, such as molecular beam epitaxy(MBE)[2] and chemical vapor deposition[1], and thus suggest further study on Raman properties of exfoliated CVT-grown $PtSe_2$ to understand this anomaly.

As for the Raman spectra of AE samples (Supplementary Figure 2b), we still observe the three prominent Raman peaks. Yet, compared to RE samples (Supplementary Figure 2c), the full width at half-maximum (FWHM) of E_g peak in AE samples broadens notably at the monolayer thickness, suggesting the material to be defective. Also, the LO peak splitting in the 3L RE sample is not observed in the 3L AE sample, possibly due to the structural disorder. Furthermore, in the E_g mode of AE samples, we observe additional peak redshift (ΔE_g), compared to that of RE samples (Supplementary Figure 2d). Similar peak red shift was observed in the E' mode of monolayer MoS_2 by sulfur vacancies[5]. Unlike bulk samples showing the overlapping spectra around E_g between RE and AE samples, we found ΔE_g increases as thickness is reduced, reaching the maximum value of ΔE_g =1.68cm^{-1} at 1L thickness (Supplementary Figure 2e). This is accompanied by a 3-fold increase in the FWHM of the peak (Supplementary Figure 2f). These features strongly indicate that ultrathin AE samples are defective.

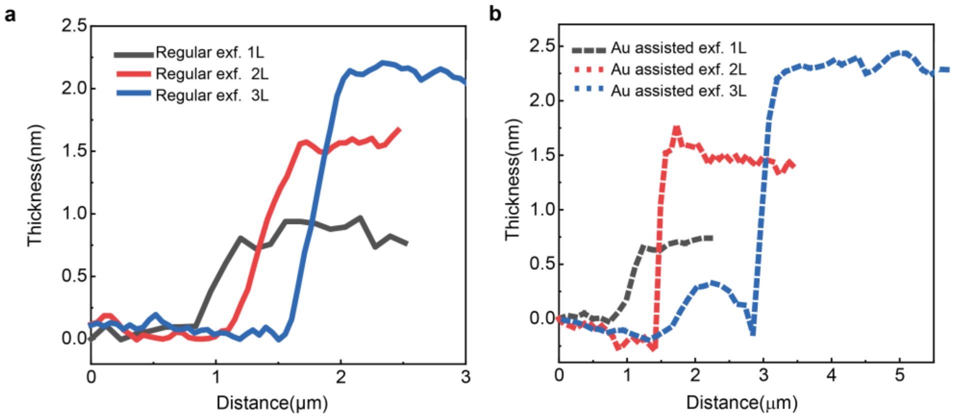

Supplementary Figure 1. Thickness of ultrathin $PtSe_2$. Mono- (1L), bi- (2L) and tri-layer (3L) $PtSe_2$ produced by RE (a) and AE were distinguished by their thickness measured by AFM.

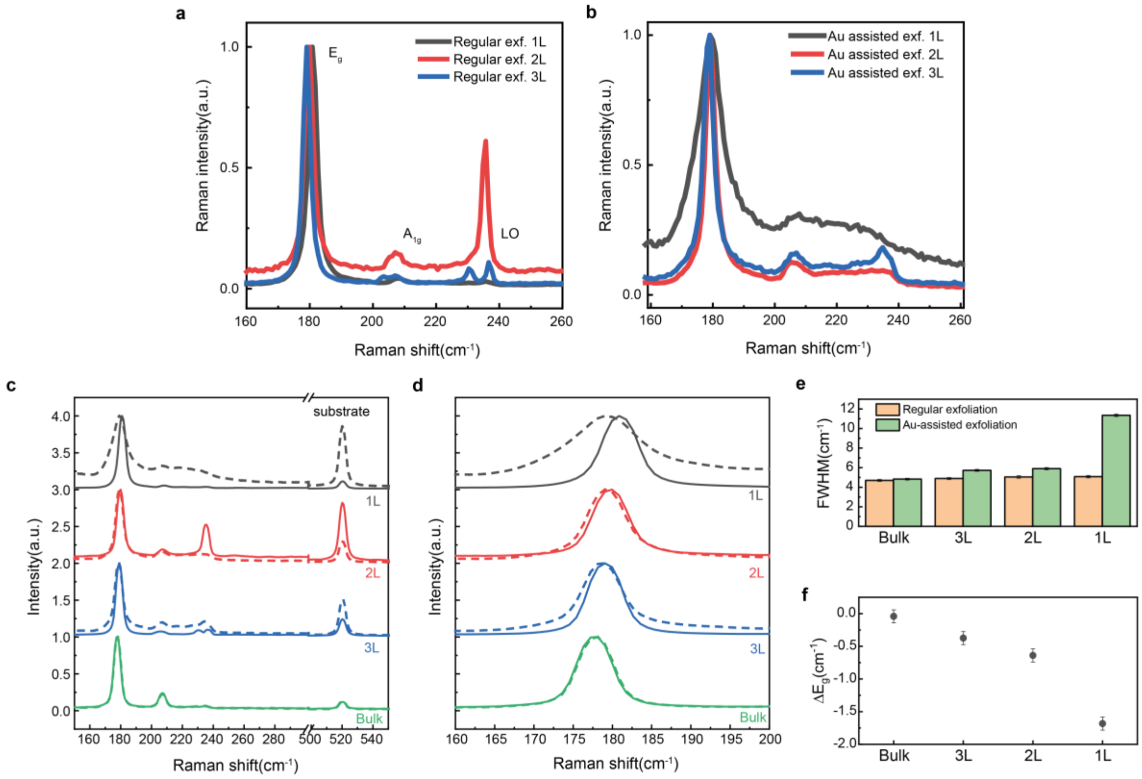

Supplementary Figure 2. Defective Raman signatures from AE samples. (a-b) Raman spectra of 1L, 2L and 3L $PtSe_2$ from RE (a) and AE (b). (c) Thickness-dependent Raman spectra compared between RE (solid line) and AE (dashed line) samples. The intensity of each Raman spectra is normalized to the intensity of its E_g mode and the spectra from 1L, 2L and 3L are given with offsets for clarity. Line colors are assigned to different layers: 1L (black), 2L (red), 3L (blue) and bulk(green). (d) Magnified spectra around E_g modes. (e) The FWHM of E_g mode compared between RE and AE samples. (f) Additional shift of E_g modes (ΔE_g) in AE samples. E_g peak positions are obtained by Lorenztian fitting, and the shift is calculated by $\Delta E_g = E_g$ (AE) - E_g (RE).

Supplementary Note 2. Raman spectroscopy on the plasma-treated RE samples

Structural damage can be created during the deposition process of Au atoms on $PtSe_2$, the effect of which would be greatest for sputtering away atoms from the very top layer of $PtSe_2$. A similar mechanical damaging process can be done by Ar-plasma treatment[6], favorably with the mild condition to etch away the top surface layer of $PtSe_2$. We have optimized the Ar-plasma etching rate by comparing the AFM-measured thickness before and after the treatment (Supplementary Figure 3a). Under the mild etching condition, we have plasma-treated the same RE samples which were examined earlier (Supplementary Figure 2a). As a result, we observe that the Raman spectra of post-treated RE samples reproduce well the Raman spectra of AE samples (Supplementary Figure 3b), supporting the idea that structural defects in AE samples are created from physical damage.

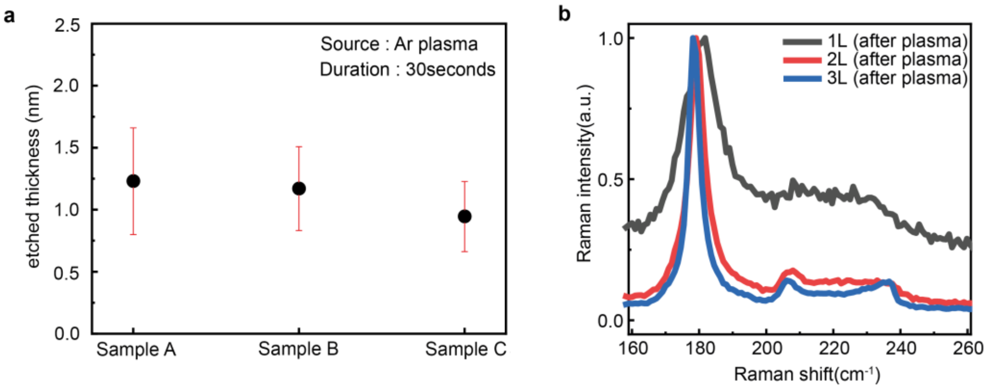

Supplementary Figure 3. Raman spectra of RE samples after mild Ar-plasma treatment. (a) Etching $PtSe_2$ by a mild Ar-plasma treatment. Mean (black dot) and standard deviation (red line) of etched thicknesses from three different $PtSe_2$ samples (A, B, and C). Ar plasma treatment effectively etches away $PtSe_2$ at the average rate of 0.36 Å/s. (b) Raman spectra of RE $PtSe_2$ sample after Ar plasma treatment with a duration of 7 seconds. At this condition, $PtSe_2$ is expected to be etched away with a nominal value of 3 Å.

Supplementary Note 3. Charge transport in bilayer $PtSe_2$

Supplementary Figure 4 shows how structural disorder in $PtSe_2$ affects the semiconducting charge transport of bilayer $PtSe_2$. Supplementary Figure 4a and Supplementary Figure 4b are the 2-terminal transfer curves from the pristine and defective devices on SiO_2/Si substrate, respectively, shown in Figure 2b of the main manuscript. While the charge transport of the pristine sample shows n-type conductance, the current in the defective sample is insensitive toward the gate voltage modulation.

The intrinsic transport properties can be better examined by encapsulating $PtSe_2$ with h-BN. Supplementary Figure 4c and Supplementary Figure 4d show the 2-terminal transfer curves from the h-BN encapsulated pristine and defective bilayer samples, respectively (see their device images from Supplementary Figure 5). Using top gate electrodes, we observe ambipolar charge transport for both samples. Yet, there is a substantial difference in the current on-off ratio whose value is 10^4 (1.4) for the pristine (defective) sample. Furthermore, a large off-state current (~30 nA at $V_{SD} = 1V$, $V_g = -2V$) is observed from the defective sample. Another notable difference is in the *I*-*V* characteristics. Supplementary Figure 4e shows the *I*-*V* curves of the pristine sample which exhibit asymmetric behavior due to Schottky barriers at the electrode/$PtSe_2$ interfaces. However, the *I*-*V* curves of the defective sample presented in Supplementary Figure 4f show a linear relationship for all the selected gate voltages. This metal-like character found in the defective sample is likely due to defect-induced mid-gap states[7].

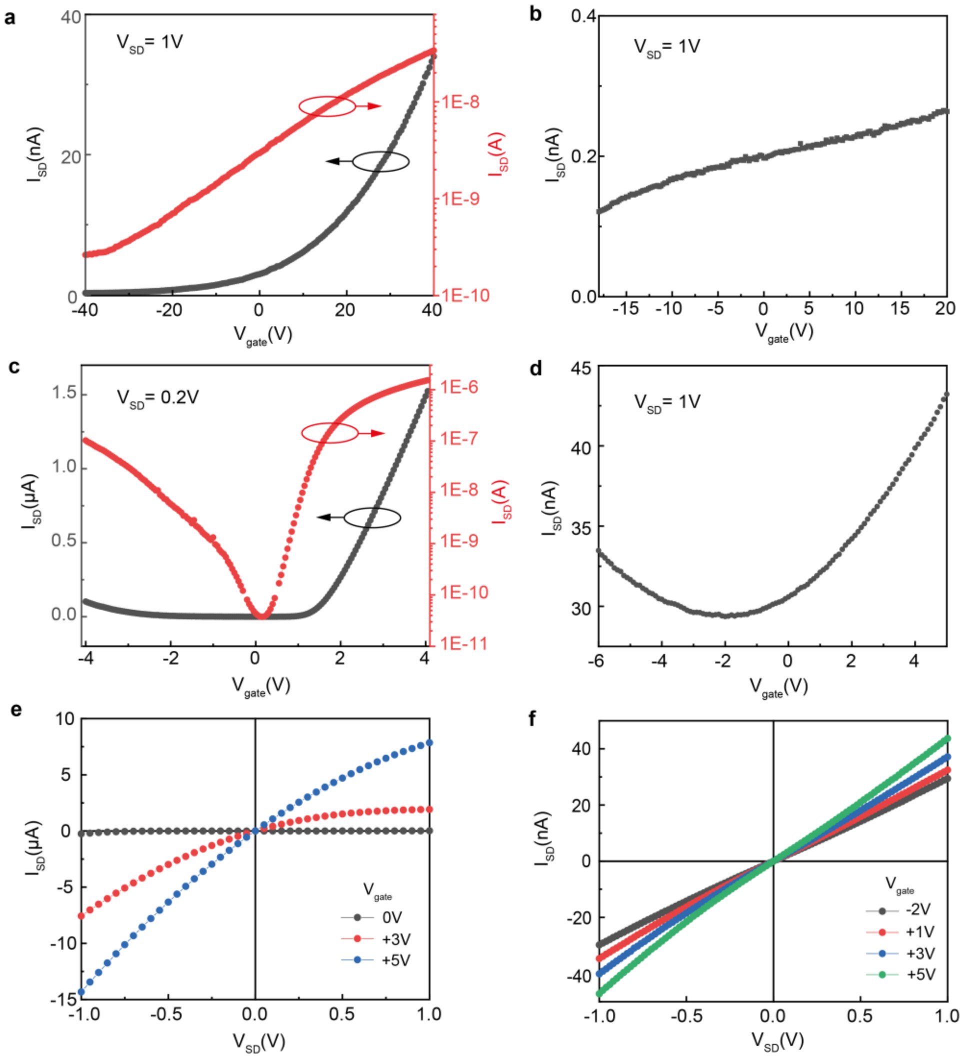

Supplementary Figure 4. Room temperature charge transport of bilayer $PtSe_2$. (a-b) Gate voltage-dependent source-drain current (I_{SD}) measured from pristine (a) and defective (b) bilayer $PtSe_2$ devices on SiO_2/Si substrate. (c-d) Gate voltage-dependent I_{SD} from h-BN encapsulated pristine (c) and defective (d) bilayer $PtSe_2$ devices. (e-f) *I-V* characteristic measured from the encapsulated pristine (e) and defective (f) bilayer $PtSe_2$ at the selected gate values.

Supplementary Note 4. Zero-biased photocurrent – polarization and spatial dependence

In the case of photocurrent measured from the zero-biased short-circuit, it shows the same characteristics as the photovoltage data introduced in the main text that photocurrent is generated from homogenous illumination of defective $PtSe_2$ (Supplementary Figure 5 and Supplementary Figure S7) and shows the concurrent linear and circular polarization dependence (Supplementary Figure 6). These properties are evidently found in h-BN encapsulated bilayer samples which showed better transport quality than the bilayer samples on a SiO_2/Si substrate (See transport result in Supplementary Figure 4).

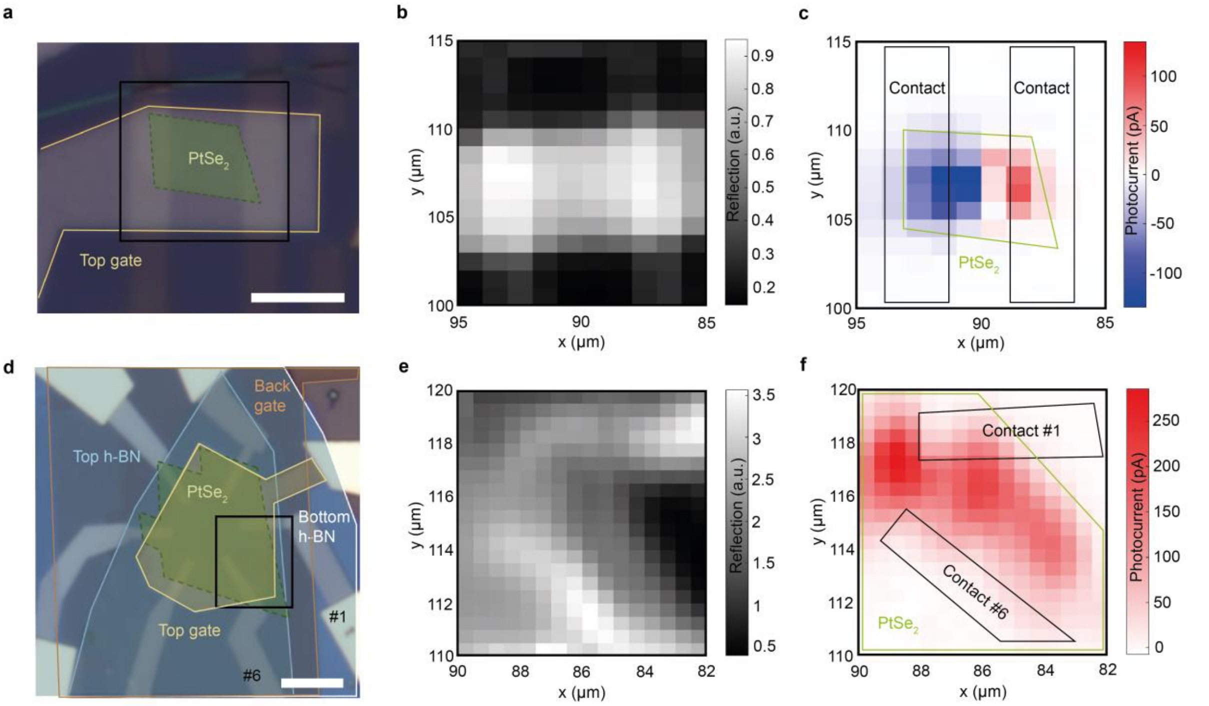

Supplementary Figure 5. Scanning photocurrent microscopy. (a-c) Optical microscopy image of h-BN encapsulated pristine 2L $PtSe_2$ device (a), its laser reflection map (b) and zero-bias photocurrent map (c) taken within the black square area in (a). (d-f) Optical microscopy image of h-BN encapsulated defective 2L $PtSe_2$ device (d), its laser reflection map (e) and zero-bias photocurrent map (f) taken within the black square area in (d). In (a) and (d), the green shaded area represents the area of 2L $PtSe_2$, and the area of the top and bottom metal gates and, h-BN layers are high-lighted in different colors. Scale bars are 5 μm. For (a), only $PtSe_2$ and top metal gate are displayed for image clarity. In (c) and (f), the position of $PtSe_2$ and contact electrodes are highlighted with green and black contour line respectively, referring to the electrode position from the laser reflection mapping.

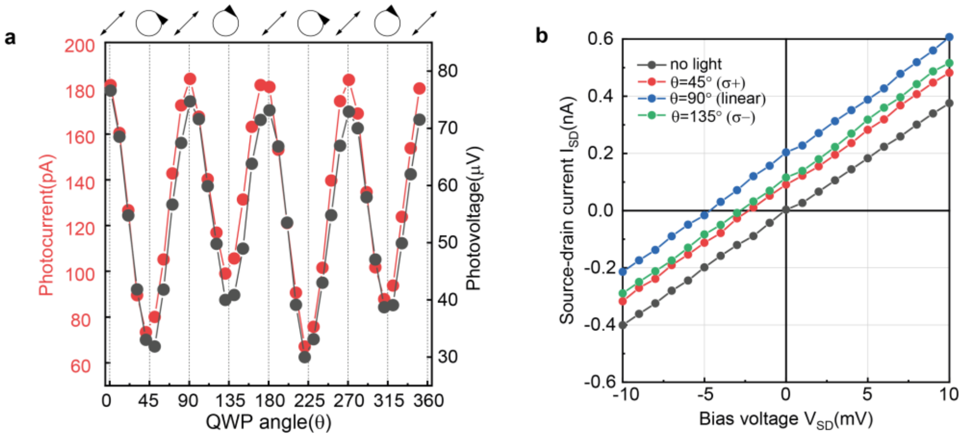

Supplementary Figure 6. Photocurrent and photovoltage showing the identical polarization dependence. (a) zero-biased photocurrent (red) and photovoltage (black) measured while rotating a quarter-wave plate angle. Here, zero-biased photocurrent is measured with DC sourcemeter at P=160μW, and photovoltage is measured with lock-in amplifier using mechanical chopper (freq.=133Hz) at P=100μW. (b) *I-V* characteristic with and without light. Linear (blue), σ+ (red), and σ- (blue) polarizations were set by different quarter-wave plate angles.

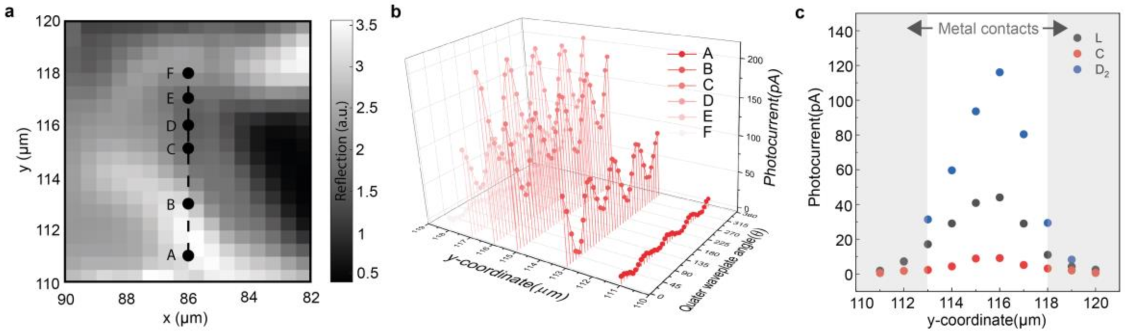

Supplementary Figure 7. Photocurrent from different illumination positions. (a) The same laser reflection map shown in **Figure S8e**, marked with laser illumination spots (A,B,…,F) between two probing metal contacts. (b) Photocurrent modulation from a quarter-wave plate rotation at different illumination spots. (c) Extracted amplitudes of linear (L), circular (C) photocurrent and the offset (D_2) along the y-coordinates from one metal contact to the other. The amplitude is averaged from the two vertical lines of x = 85 μm, and 86 μm in (a). The positions of metal contacts are grey-shaded. The amplitude of all the components increases maximally at the center position of two probing contacts.

Supplementary Note 5. Optical power *vs.* zero-biased photocurrent

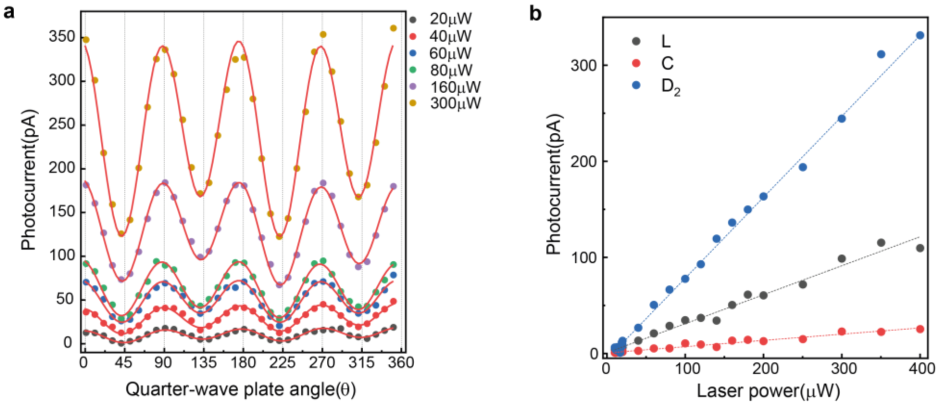

Supplementary Figure 9. Laser power dependence. (a) Quarter-wave plate angle-dependent zero-biased photocurrent measured at different laser power. The red curve is the fitting equation (Eq. 1 from the main manuscript). (b) Extracted amplitudes of linear (L), circular (C) photocurrent and the offset (D_2) with respect to the laser power. All the amplitudes show linear power dependence fitted by linear dashed lines.

Supplementary Note 6. Anisotropic photocurrent from *ab initio* calculation

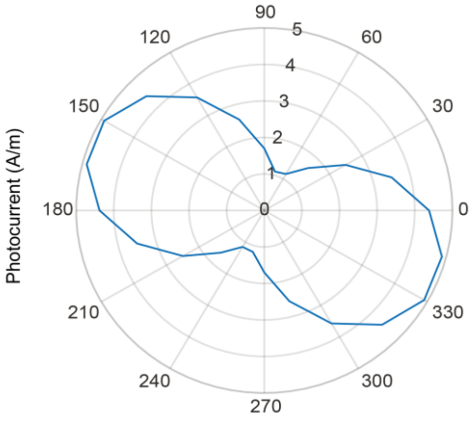

Supplementary Figure 10. Calculated photocurrent showing anisotropic linear polarization dependence. Here, 0°. corresponds to armchair direction of $PtSe_2$.

Supplementary Note 7. Phenomenological LPGE equation based on the point symmetry of V_{Se}

We start with taking into consideration of Neumann's principle of symmetry which states that the symmetry of any physical property of a crystal must include the symmetry elements of the point group of the crystal[8]. The relevant physical property to LPGE is the second-order susceptibility tensor and, without considering the microscopic origin, the photocurrent generated by LPGE (J^{LPGE}) can be written as[9],

$$J_{\alpha}^{LPGE} = \chi_{\alpha\beta\gamma}\left(E_{\beta}(\omega)E_{\gamma}^{*}(\omega) + E_{\beta}(\omega)E_{\gamma}^{*}(\omega)\right). \tag{S1}$$

Here, E is the electric field and χ is the third-rank photogalvanic tensor. α, β and γ runs over all the Cartesians coordinates (x, y and z).

When the inversion operation is applied to Eq. (S1), J_{α}^{LPGE} changes its sign, yet $E_{\beta}(\omega)E_{\gamma}^{*}(\omega)$ does not and χ maintains the sign under the inversion operation for $PtSe_2$. To satisfy the sign of the equation, all the elements in χ has to be zero, which, in order words, LPGE is not allowed for $PtSe_2$. However, our experimental observation points out χ should have none-zero elements. We consider the term, crystal, noted in Neumann's principle to be instead defective $PtSe_2$ with Se vacancies. The choice of Se vacancies among other structural defects is based on the following arguments:

1. For 2D TMDCs, comparatively low threshold energy is required to create chalcogen vacancies[10].
2. Photocurrent by *ab initio* calculation with Se vacancies matches well with our experimental data (compare Figure4c and Supplementary Figure 10).
3. The presence of Se vacancies from the sample studied in this work is confirmed by scanning tunnelling electron microscopy image (See **Figure1c** in the main manuscript).

The local symmetry of Se-vacancy restricts the form of the second-order susceptibility tensor. As shown in Supplementary Figure 11a, there are three mirror planes (M_{y-z}, M_{y-z}', and M_{y-z}''), perpendicular to the xy plane and 3-fold rotation axis located at the missing Se site, constructing C_{3v} point group symmetry for Se vacancy. For this point group, there are four linearly independent components in the χ[11]:

$$\chi_1 = \chi_{xxz} = \chi_{yyz}, \tag{S2}$$

$$\chi_2 = \chi_{xxy} = \chi_{yxx} = -\chi_{yyy}, \tag{S3}$$

$$\chi_3 = \chi_{zxx} = \chi_{zyy}, \tag{S4}$$

$$\chi_4 = \chi_{zzz}, \tag{S5}$$

where the x-axis is defined as the direction perpendicular to the M_{y-z} mirror plane. We note that x-axis is along one zig-zag direction of $PtSe_2$. Inserting Eq. (S2-5) into Eq. (S1) gives,

$$\begin{bmatrix} J_x \\ J_y \\ J_z \end{bmatrix} = \begin{bmatrix} 0 & 0 & 0 & 0 & \chi_1 & -\chi_2 \\ -\chi_2 & \chi_2 & 0 & \chi_1 & 0 & 0 \\ \chi_3 & \chi_3 & \chi_4 & 0 & 0 & 0 \end{bmatrix} \begin{bmatrix} E_x^2 \\ E_y^2 \\ E_z^2 \\ 2E_yE_z \\ 2E_xE_z \\ 2E_xE_y \end{bmatrix}. \tag{S6}$$

As we are interested in photoresponse generated in the 2D plane, we only consider J_x and J_y. Below, we use the notation of x_0, y_0 and z_0 instead for x, y and z Cartesians coordinate for the sake of convenience for the later explanation.

From Eq. (S6), J_{x0} can be written as

$$J_{x0}^{LPGE} = \chi_1(E_{x0}E_{z0}^* + E_{z0}E_{x0}^*) + \chi_2(E_{x0}E_{y0}^* + E_{y0}E_{x0}^*) \quad \text{(S7)}$$

and J_{y0} can be written as

$$J_{y0}^{LPGE} = \chi_1(E_{y0}E_{z0}^* + E_{z0}E_{y0}^*) + \chi_2\left(|E_{x0}|^2 - |E_{y0}|^2\right). \quad \text{(S8)}$$

Considering normal incidence ($E_{z0} = 0$), Eq. (S7) becomes

$$J_{x0}^{LPGE} = \chi_2(E_{x0}E_{y0}^* + E_{y0}E_{x0}^*), \quad \text{(S9)}$$

and Eq. (S8) becomes,

$$J_{y0}^{LPGE} = \chi_2\left(|E_{x0}|^2 - |E_{y0}|^2\right). \quad \text{(S10)}$$

Consider the angle α_0 of electric field from x_0, Eq. (S9) and Eq. (S10) can be respectively re-written as

$$J_{x0}^{LPGE} = \chi_2(E_{x0}E_{y0}^* + E_{y0}E_{x0}^*) = \chi_2|E_0|^2 2sin2\alpha_0, \quad \text{(S11)}$$

and

$$J_{y0}^{LPGE} = \chi_2\left(|E_{x0}|^2 - |E_{y0}|^2\right) = \chi_2|E_0|^2 2cos2\alpha_0. \quad \text{(S12)}$$

Here, setting a coordinate axis parallel to the initial E field direction, that is set by the initial alignment of the fast axes of linear polarizer and half-wave plate is more useful from the experimental point of view. We rotate the coordinate with arbitrary $-\varphi$ wrt. to x_0-axis and make new x and y coordinates, the schematic of which is illustrated in Supplementary Figure 12a.

Defining $\alpha_0 = \alpha - \varphi$, Eq. (S11) becomes

$$J_{x0}^{LPGE} = \chi_2|E_0|^2 2sin2\alpha_0 = \chi_2|E_0|^2 2sin2(\alpha - \varphi), \quad \text{(S13)}$$

and Eq. (S12) becomes

$$J_{y0}^{LPGE} = \chi_2|E_0|^2 2cos2\alpha_0 = \chi_2|E_0|^2 2cos2(\alpha - \varphi). \quad \text{(S14)}$$

Then, we re-write them in the rotated coordinates. The photocurrent generated in the x direction is

$$J_x^{LPGE} = J_{x0}^{LPGE}\cos\varphi - J_{y0}^{LPGE} sin\,\varphi. \quad \text{(S15)}$$

Inserting Eq. (S13) and Eq. (S14), Eq. (S15) becomes

$$J_x^{LPGE} = \chi_2|E_0|^2 2sin2(\alpha-\varphi)\cos\varphi - \chi_2|E_0|^2 2cos2(\alpha-\varphi)\sin\varphi \\ = \chi_2|E_0|^2 sin(2\alpha - 3\varphi). \quad \text{(S16)}$$

Similarly, the photocurrent generated in the y direction is

$$V_y^{LPGE} = V_{x0}^{LPGE}\ \sin\varphi + V_{y0}^{LPGE}\cos\ \varphi, \quad \text{(S17)}$$

which is

$$J_y^{LPGE} = \chi_2|E_0|^2 2sin2(\alpha-\varphi)\sin\varphi + \chi_2|E_0|^2 2cos2(\alpha-\varphi)\cos\varphi \\ = \chi_2|E_0|^2 cos(2\alpha - 3\varphi). \quad \text{(S18)}$$

Eq. (S16) and Eq. (S18) show that the amplitude and the direction of photocurrent are determined by electric field of light and crystallographic direction.

Now we consider the contact geometry for measuring LPGE. The signal measured by a contact is achieved by orthogonal projection of J_x^{LPGE} and J_y^{LPGE} along the direction of the contact. If we consider the position of a pair of electrodes for 2 terminal measurement as shown in Supplementary Figure 12b, which is rotated by κ_1 with respect to the x-axis, then the photocurrent measured along these electrodes (J^1) is

$$J^1 = J_x^{LPGE}\cos\kappa_1 + J_y^{LPGE}\sin\kappa_1. \quad \text{(S19)}$$

Inserting Eq. (S16) and Eq. (S18) into Eq. (S19), J^1 can be expressed as

$$J^1 = \chi_2|E_0|^2(\sin(2\alpha-3\varphi)\cos(\kappa_1) + \cos(2\alpha-3\varphi)\sin(\kappa_1)), \quad \text{(S20)}$$

and more simply,

$$J^1 = \chi_2|E_0|^2\sin(2\alpha - 3\varphi + \kappa_1). \quad \text{(S21)}$$

We can write Eq. (S21) in a general form describing the photocurrent measured from the n-pairs of electrodes, which is

$$J^n = \chi_2|E_0|^2\sin(2\alpha - 3\varphi + \kappa_n). \quad \text{(S22)}$$

The photovoltage measured under open-circuit condition can be described with a proportional coefficient (t) and can be written as,

$$V^n = t\chi_2|E_0|^2\sin(2\alpha - 3\varphi + \kappa_n). \quad \text{(S23)}$$

From Eq. (S22), we can extract the phase of each data as $3\varphi - \kappa_n$, which consists of φ, the relative angle of zig-zag direction of $PtSe_2$, and κ_n, the relative angle between nth contact from the initial direction of the applied electric field.

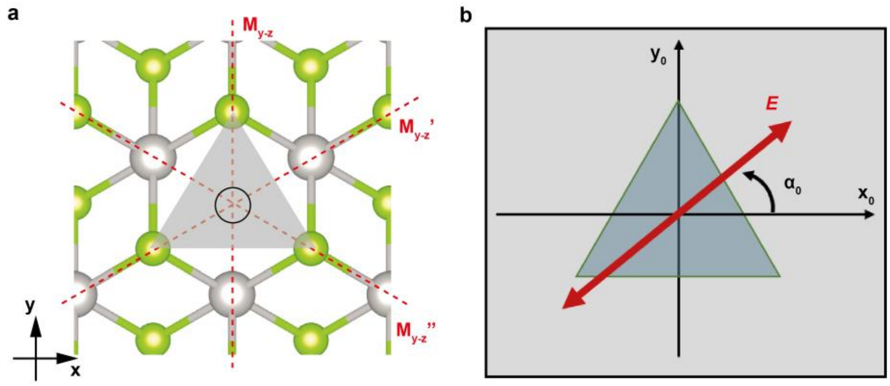

Supplementary Figure 11. Se vacancy as an atomic site for wedge potential. (a) Schematic representation of Se vacancy in $PtSe_2$ and its point group symmetry (C_{3v}). (b) Schematic of a wedge potential. The x_0-axis is the axis perpendicular to the mirror plane of the wedge, parallel to y_0-axis. A double red arrow indicates the electric field from the incident light. The azimuth angle α_0 is counted from the x_0 axis.

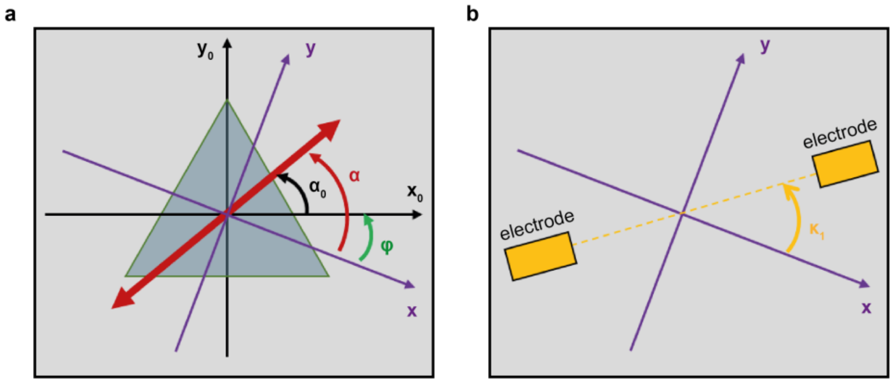

Supplementary Figure 12. LPGE with experimentally accessible coordinates. (a) Schematic representation of new x- and y-axes tilted by an angle φ with respect to the x_0 and y_0 axes. The azimuth angle is redefined as α counted from the x-axis. Here, the x-axis belongs to the linear polarization of light at α=0. (b) Schematic representation of a diagonal contact pair positioned in an angle of κ_1 counted from the x-axis.

Supplementary Note 8. Extracting electrode angles from LPGE equation

Here, we demonstrate the validity of Eq. (S23) by fitting the measured data of Figure 4d in the main manuscript with Eq. (S23) (Supplementary Figure 13a). Since we do not know the crystallographic orientation of $PtSe_2$, for the moment, we use arbitrary input $\varphi=0$ for the fitting to extract the electrode angles (κ_n). The electrode angles, which are extracted from the fitting of data measured from 1st to 6th diagonal electrode pairs, increases with a 61deg. step on average **(Supplementary Figure 13b**). This increment describes well the 6-fold symmetry of the electrode geometry shown in Figure 4b in the main manuscript. We note that the increment steps are fixed for other φ fitting parameters and hence φ only changes the offset of the extracted electrode angles.

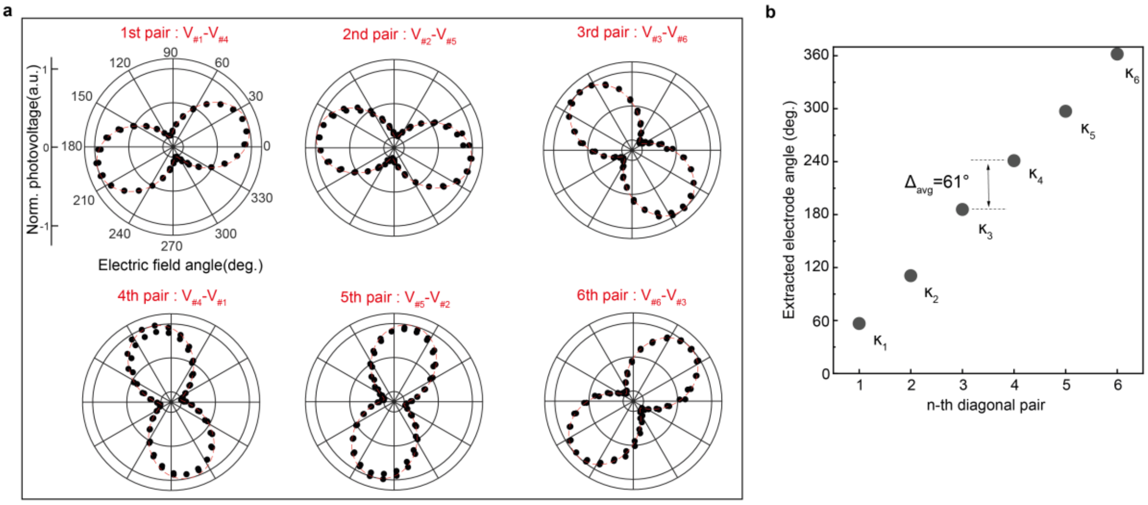

Supplementary Figure 13. Data fitting with Eq. (S22). (a) Normalized photovoltage polar plots from six diagonal pairs with a sinusoidal fit (red dash line) using the normalized model equation, $V^n_{Norm} = \sin(2\alpha - 3\varphi + \kappa_n)$. Data (black dots) are measured from one full rotation of the half-wave plate. b) Electrode angles (κ_n) extracted by an arbitrary input parameter of φ (φ=0).

Supplementary Note 9. Determining electrode angles and zig-zag directions of $PtSe_2$

For measuring the data of Figure 4d shown in the main manuscript, we aligned both the fast axes of linear polarizer and HWP along one movement axis of XY nano-positioner, which is in parallel to the x-axis of a 2D reflection mapping of the device (Supplementary Figure 14a). With the initial linear polarization (HWP angle = 0deg.) set to x-axis of this image, we deduced the angle k_n of nth contact electrodes $(n = 1,2, \ldots 6)$ with respect to the x-axis (Supplementary Figure 14b), which is close to $k_n = 9 + (n-1) \times 60$ degrees, neglecting their decimal values. The angles are in anti-clockwise following the anti-clockwise rotation of HWP.

Using this set of k_n, the angle of zig-zag direction is extracted to be 43.7 degrees (Figure 4e in the main manuscript). The angle of the other zig-zag directions can be calculated based on their 3-fold symmetry. The three zig-zag directions (φ_1, φ_2 and φ_3) are overlaid on top of reflection mapping with a reference line connecting electrode #1 and #4 (Supplementary Figure 14c). Using this reference line, we have compared the extracted zig-zag directions to the sample edges (Figure 4f in the main manuscript).

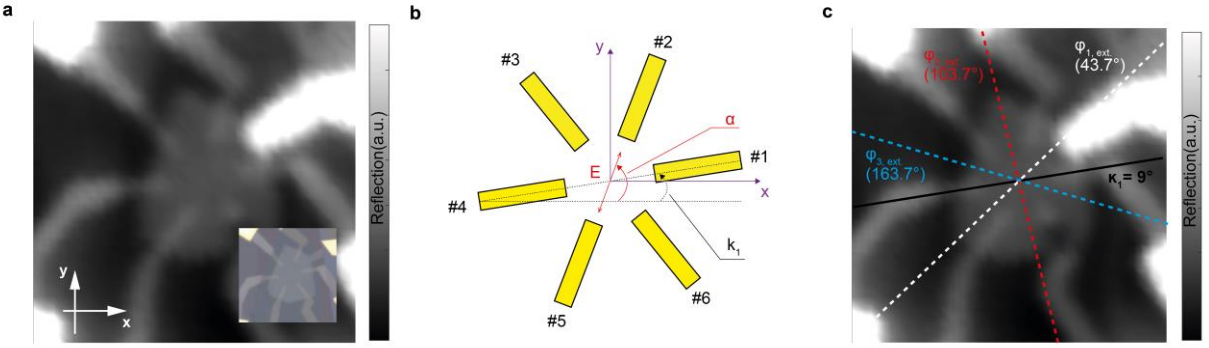

Supplementary Figure 14. Determining the angle of contact electrodes and $PtSe_2$ zig-zag directions. (a) 2D laser reflection map of the device with circularly-oriented electrodes. Inset shows the optical microscopy image of the device. The initial linear polarization of light is aligned in parallel to the x-axis. (b) Schematic of contact electrode alignments. The nth contact electrode (n=1,2…6) is positioned at anti-clockwise angle with respect to the x-axis. α and k_1 are defined as the angle of linear light polarization and the angle of #1 electrode with respect to the x-axis, respectively. (c) The extracted 1st, 2nd and 3rd zig-zag directions (white, red and blue dash lines, respectively) overlaid on the reflection map. The black line is a reference line connecting electrode #1 and electrode #4.

Supplementary Note 10. EDX analysis

EDX spectroscopy was performed on multilayer $PtSe_2$ samples to investigate if there are any remaining Au layers/atoms on $PtSe_2$ from the Au-assisted exfoliation method. The low characteristic X-ray emissions from the two samples (regular exfoliation and Au-assisted exfoliation) on a SiO_2/Si substrate are first compared (

Supplementary Figure **15**a). However, it is difficult to argue the presence or absence of Au from the low X-ray emission range as the characteristic peak (M_α) of Au and Pt are energetically close to each other (Pt M_α: 2.048eV, Au M_α: 2.120eV). The higher characteristic peaks, $L_{\alpha1}$ emission of Pt and Au, are more energetically apart (Pt $L_{\alpha1}$: 9.442eV, Au $L_{\alpha1}$: 9.713eV). From the higher X-ray emission range enabled by higher electron acceleration voltage, we only observe the $L_{\alpha1}$ emission of Pt from the sample obtained by Au-assisted exfoliation (

Supplementary Figure **15**b). Hence, we can say that the content of Au is negligible or below the EDX detection limit, for $PtSe_2$ from the Au-assisted exfoliation method.

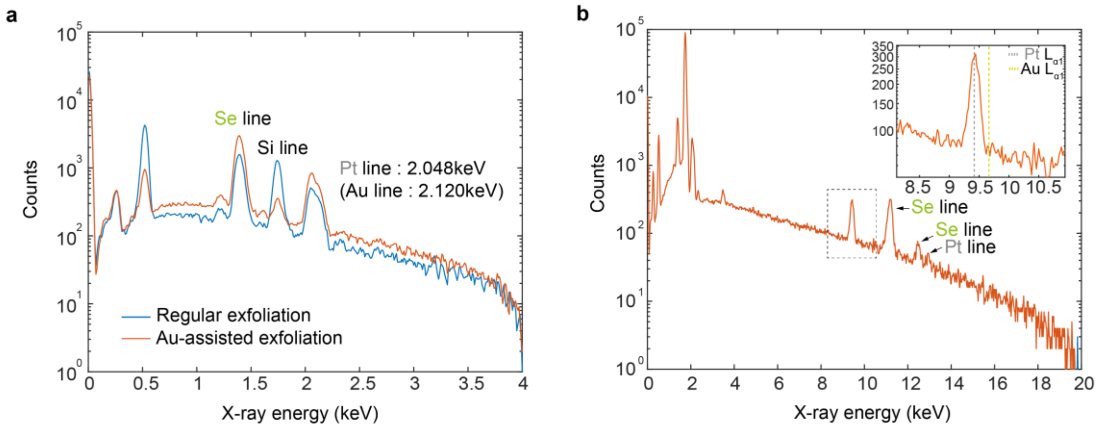

Supplementary Figure 15. EDX spectroscopy on $PtSe_2$ from Au-assisted exfoliation. (a) low energy range. (b) high energy range. The inset shows magnified view for the data marked by a dotted square.

References

1. Gulo, D. P., Yeh, H., Chang, W.-H. & Liu, H.-L. Temperature-dependent optical and vibrational properties of $PtSe_2$ thin films. *Sci. Rep.* **10**, 19003 (2020).
2. Yan, M. *et al.* High quality atomically thin $PtSe_2$ films grown by molecular beam epitaxy. *2D Mater.* **4**, 045015 (2017).
3. O'Brien, M. *et al.* Raman characterization of platinum diselenide thin films. *2D Mater.* **3**, 021004 (2016).
4. Lee, C. *et al.* Anomalous Lattice Vibrations of Single- and Few-Layer MoS_2. *ACS Nano* **4**, 2695–2700 (2010).
5. Parkin, W. M. *et al.* Raman Shifts in Electron-Irradiated Monolayer MoS_2. *ACS Nano* **10**, 4134–4142 (2016).
6. Liu, Y. *et al.* Layer-by-Layer Thinning of MoS_2 by Plasma. *ACS Nano* **7**, 4202–4209 (2013).
7. Shawkat, M. S. *et al.* Thickness-Independent Semiconducting-to-Metallic Conversion in Wafer-Scale Two-Dimensional $PtSe_2$ Layers by Plasma-Driven Chalcogen Defect Engineering. *ACS Appl. Mater. Interfaces* **12**, 14341–14351 (2020).
8. Neumann, F. E. & Meyer, O. E. Vorlesungen über die Theorie der Elasticität der festen Körper und des Lichtäthers. (Teubner, 1885).
9. Sturman, B. I. & Fridkin, V. M. *The Photovoltaic and Photorefractive Effects in Noncentrosymmetric Materials*. (Routledge, 2021). doi:10.1201/9780203743416.
10. Komsa, H.-P. *et al.* Two-Dimensional Transition Metal Dichalcogenides under Electron Irradiation: Defect Production and Doping. *Phys. Rev. Lett.* **109**, (2012).
11. Boyd, R. W. Chapter 1 - The Nonlinear Optical Susceptibility. in *Nonlinear Optics (Fourth Edition)* (ed. Boyd, R. W.) 1–64 (Academic Press, 2020). doi:10.1016/B978-0-12-811002-7.00010-2.
